# Alcohol and the Developing Brain: Neuroanatomical Studies

**Published:** 2003

**Authors:** Wei-Jung A. Chen, Susan E. Maier, Scott E. Parnell, James R. West

**Affiliations:** Wei-Jung A. Chen, Ph.D., is an assistant professor; Susan E. Maier, Ph.D., is a research assistant professor; Scott E. Parnell is a graduate student; and James R. West, Ph.D., is a distinguished professor; all in the Department of Human Anatomy and Medical Neurobiology, College of Medicine, Texas A&M University System Health Science Center, College Station, Texas

**Keywords:** prenatal alcohol exposure, neuroimaging, neurotoxicity, fetal alcohol effects, brain damage, cognitive development, AODR (alcohol and other drug related) behavioral problem, prevention strategy, drug therapy, CNS function, cell adhesion molecules, mitochondria

## Abstract

One of the distinguishing features of prenatal alcohol exposure is impaired cognitive and behavioral function resulting from damage to the central nervous system. Information available from the small number of autopsied cases in humans indicates that the offspring of mothers who abused alcohol during pregnancy have various neuroanatomical alterations ranging from gross reductions in brain size to cellular alterations. Recent neuroimaging technology provides the most powerful tool for assessing the neurotoxic effects of fetal alcohol exposure in living organisms and for exploring the relationship between behavioral dysfunction and brain damage at the regional level. Recently, animal research has suggested that the damaging effects of alcohol exposure during brain development could be prevented or attenuated by various pharmacological manipulations or by complex motor training. These promising findings provide directions for developing future prevention or intervention strategies.

Prenatal alcohol exposure adversely affects the developing anatomical structures of the body and brain, leading to a range of physical, cognitive, and behavioral effects. The term “anatomical structures” encompasses both the components of the major body systems (e.g., heart, blood vessels, bones, muscles) and the cellular and molecular structures within these major components. Any alterations to the body’s anatomical structures, regardless of the level at which they occur (gross or microscopic), may negatively affect an organism’s function. This article reviews changes in brain anatomy (i.e., neuroanatomy) that occur following developmental (prenatal and/or early postnatal) alcohol exposure in both humans and animal models. It also discusses promising techniques to prevent or reverse alcohol-induced neuroanatomical changes.

The most serious consequence of prenatal alcohol exposure is a constellation of symptoms known as fetal alcohol syndrome (FAS). The criteria for diagnosing FAS include facial dysmorphology, growth retardation, and central nervous system (CNS) dysfunction. Facial dysmorphology results from anatomical changes occurring during weeks 4 to 8 of gestational development that affect how tissues merge under the facial prominences. Of all anomalies associated with FAS, growth retardation is the most obvious form of anatomical change. People who are not professionally trained to analyze facial dysmorphology can easily recognize this anomaly because the weight and size of FAS infants are distinctly smaller than normal. Anatomical alterations associated with CNS dysfunction may range from gross reduction in brain volume (i.e., microencephaly), to deficits in cell number in a particular brain region, to cellular modifications of individual nerve cells (i.e., neurons), to alterations in the communications among cells. These alterations can have a long-term detrimental impact on behavioral and cognitive development.

## Human Neuroanatomical Studies

Since FAS was defined several decades ago, researchers have learned a significant amount about the nature of the behavioral, cognitive, and physical features of FAS and related diagnoses. Until recently, not much was known about the actual anatomical injury to the brains of children with FAS and the relationships between risk factors and behavioral/cognitive outcomes and brain injury. This information could only be acquired at autopsy, and because FAS generally is not life threatening, there were few cases available for study. Researchers had to use caution when reviewing the autopsy data derived from children with FAS who died for reasons other than accidents because brain tissues may be severely affected by other life-threatening diseases. Therefore, researchers had to rely on animal models to address most of these questions. Now that magnetic resonance imaging (MRI) and functional MRI (fMRI) are available for use with living subjects, researchers are generating new data that can lead to a better understanding of brain and behavior relationships in humans, as well as a better interpretation of findings from animal studies. This new information will guide researchers working with animals in their attempts to target mechanisms and potential therapeutic interventions for humans.

### Microcephaly

Microcephaly is defined as having a small head relative to body size and is based on the ratio of body weight to head circumference or height**-**to**-**head circumference (not to be confused with microencephaly, which refers to the size of the *brain*). Early studies showed that microcephaly was related to alcohol use throughout pregnancy (Ernhart et al. 1985; Rosett et al. 1983). Coles (1994) reported that children whose mothers stopped drinking before the end of the second trimester had larger head circumferences on average than children whose mothers continued to drink throughout pregnancy. This finding suggests that a pregnant woman may be able to avoid additional injury to her baby’s brain if she stops drinking before the third trimester. These data are useful for counseling pregnant women about the benefits to their unborn children of reducing or ceasing their alcohol consumption as soon as their pregnancy is identified.

### Neuroimaging

In recent years, researchers have used both anatomical MRI and functional MRI to determine the nature of brain injury in living offspring with FAS. Early seminal MRI studies by Riley and colleagues (reviewed by Roebuck et al. 1998) showed reductions in the volume of several brain regions in children who were affected by heavy^1^ maternal drinking. These studies were significant because they showed a reduction in the size of specific brain regions independent of any reduction in total brain volume.

More recently, Archibald and colleagues (2001) found that, in children with FAS, the fibers carrying information between brain cells (i.e., white matter) appear to be more susceptible to reductions in volume than the outer, convoluted layer of brain tissue called the cerebral cortex. Within the cerebral cortex, the parietal lobe (situated at the upper middle of the cerebrum) is reduced by prenatal alcohol exposure, whereas the temporal and occipital lobes (located on the sides and back of the brain, respectively) are not. These anatomical data clearly show that alcohol’s effect on the developing brain is not uniform but varies depending on the brain region. Wass and colleagues (2001) used ultrasound sonography to examine living fetuses exposed to alcohol and found a reduction in the size of the frontal cortex, but not other cortices, of the cerebrum.

Clark and colleagues (2000) used MRI to examine the brains of adults who had been exposed to alcohol in utero and were classified as nonretarded (as assessed by IQ score). They found only one abnormal case, a thinning corpus callosum in the adult with the lowest IQ. (The corpus callosum is a bundle of fibers through which the two cerebral hemispheres communicate.) Otherwise, the MRIs of all other study participants were considered to be in the normal range. Some of the participants from this study were examined further using positron emission tomography (PET). These scans revealed changes in metabolic rate in the thalamus and the head of the caudate nucleus (two brain structures important for communication within the brain) (Clark et al. 2000). The authors suggest that the functional findings may be related to the regional vulnerability of the brain to prenatal alcohol exposure, which is consistent with the conclusion of an earlier PET study (Hannigan et al. 1995).

More recently, Bookstein and colleagues (2002a, b) explored the relationship between the shape of the corpus callosum in adolescents and adults (assessed with MRI) exposed to alcohol prenatally and the degree of their cognitive impairment, to see if the shape of the corpus callosum was an indicator of heavy in utero alcohol exposure. Although the volume of the corpus callosum in the alcohol-exposed study participants was not different from that in the control participants, the variability in the shape of the corpus callosum was much higher in the alcohol-exposed group. The authors suggest that variations in corpus callosum size, volume, and shape revealed by MRI can be used as diagnostic criteria for FAS and related brain disorders. Thus, MRI could be used to solicit more social support services, which often are lacking for adolescents and adults affected by prenatal alcohol exposure.

### Autopsy Studies

Anatomical changes occurring at the cellular level in humans can best be revealed with autopsy material. Although only a very small number of brains are available for this level of analysis, finding any microscopic changes in the brain lends considerable support to the claim that developmental alcohol exposure induces neuronal injury, which ultimately may be responsible for the behavioral and cognitive changes observed in children exposed to alcohol prenatally.

Clarren and associates (1978) found changes in the structure of the autopsied brains of four children diagnosed with FAS. The most prevalent findings in these brains were incorrectly located (i.e., ectopic) neurons in the white matter, suggesting errors in the migration of neurons throughout the brain. In one case there was a complete absence of the corpus callosum and anterior commissure, another fiber bundle that connects regions of the brain. Ferrer and Galofre (1987) found that the short, hair-like projections (i.e., dendritic spines) on fibers called dendrites, which extend from neurons to receive information from other neurons, also were affected. They reported that the brain from a 4-month-old infant with FAS showed a significant decrease in the number of dendritic spines on nerve cells projecting from the cortex to other parts of the brain and spinal cord. Another autopsy case, of a 2-month-old infant born to a mother who binge drank during pregnancy (primarily during the first trimester), revealed absent olfactory bulbs and tracts (which the brain uses to sense odors); poorly developed optic tracts; fused anterior brain structures such as the septum, thalamus, and the head of the caudate nucleus; fewer cells in the dentate gyrus, which is part of the hippocampus (an area crucial for memory); and fewer nerve cells in the cerebellum (which regulates balance, posture, movement, and muscle coordination) known as Purkinje cells, and disorientation of these cells (Coulter et al. 1993). Although these autopsy data are based on a small number of subjects, they seem to show that prenatal alcohol exposure does have a damaging effect on the structural organization of the developing human brain.

## Animal Neuroanatomical Studies

Most of the current information on how developmental exposure to alcohol induces brain damage comes from animal studies. Because of the obvious ethical constraints of performing certain procedures on humans, the retrospective nature of the human data, and numerous confounding variables, human studies cannot address issues that can be addressed with animal studies. Researchers are able to control factors such as dose, timing, and pattern of alcohol exposure in animal studies. And because there are so few human autopsy cases, researchers have performed most neuroanatomical assessments, particularly at the cellular level, on animals. The following sections briefly summarize several hallmark findings related to neuroanatomical studies of the effects of developmental alcohol exposure in animals.

### Microencephaly

Microencephaly, defined in animal subjects as having a small brain relative to body size, is a gross neuroanatomical anomaly associated with heavy alcohol exposure during development. As described above, such an anomaly in humans is inferred by deficits in head circumference because we cannot remove and weigh the human brain. However, in animals, the most common and traditional means of measuring microencephaly is brain weight.

Numerous reports have shown that alcohol exposure during the brain growth spurt (a period of most intense brain growth) in rats significantly reduces the weight of the forebrain, brain stem, and cerebellum (Maier et al. 1997). Importantly, in the microencephalic brain, not every brain region is affected equally. Using state-of-the-art three-dimensional stereological techniques, researchers can estimate the volume (size) of various brain regions to demonstrate microencephaly. Many of these stereological studies have demonstrated that developmental alcohol exposure leads to reductions both in brain weight and volume in a rat model (Maier et al. 1997; Chen et al. 1998). It appears that heavy alcohol exposure during the brain growth spurt (early postnatal period in rats, and third trimester and early infancy in humans) leads to the most severe and pronounced microencephaly compared with exposure during other developmental stages. Surprisingly, in rats, even 1 day of alcohol exposure at a high dose (6.6 grams per kilogram body weight) is sufficient to cause growth deficits in specific brain regions, such as the cerebellum (Goodlett et al. 1990). Olney and colleagues (2002) recently have suggested that these alcohol-induced reductions in brain volume and neuronal loss may be attributed to alcohol-induced neurodegeneration via programmed cell death (i.e., apoptosis).

### Neuronal Loss

One of the most devastating and extreme consequences of developmental alcohol exposure is the loss of neurons, and the data documenting neuronal loss are derived mostly from animal studies. Although selective programmed neuronal death is a normal aspect of CNS development, excessive neuronal death disrupts the development of normal neural networks and may lead to cognitive and behavioral dysfunctions (in both humans and animals). Distinct regions and specific cell types appear to be affected by alcohol-induced neuronal loss. Studies in rats have clearly demonstrated that alcohol causes a reduction of cerebellar Purkinje cells, cells of the olfactory bulb, and pyramidal cells in a part of the hippocampus known as the CA1 region (Chen et al. 1998, 1999*a*; Livy et al. 2003). In other neuronal populations, however, such as those in the ventrolateral nucleus of the thalamus (an area important for voluntary movement) and the locus coeruleus (a group of nerve cells involved in arousal and vigilance), alcohol does not cause cell loss (Chen et al. 1999*b*; Livy et al. 2001). This pattern of alcohol-induced loss of neurons is important for two reasons: (1) it establishes a correlation between the severity of neuronal loss of a specific neuronal population and the degree of behavioral deficits associated with such a cell population, and (2) it provides insight into the specific characteristics of neuronal populations that are most vulnerable or most resistant to alcohol-induced neuronal loss.

### Fewer Dendritic Spines

Proper CNS communication depends on an adequate number of dendritic spines and dendritic branching, because dendrites are the sites of contact for neurons. Animal studies, both in vivo and in vitro, have demonstrated that developmental alcohol exposure negatively affects the structural integrity of the dendrites and the number of dendritic spines in neurons located in the substantia nigra (a group of nerve cells involved in movement), the cortex, and the hippocampus (Tarelo-Acuna et al. 2000; Yanni and Lindsley 2000; Shetty et al. 1993; Fabregues et al. 1985). For example, Tarelo-Acuna and colleagues (2000) showed that prenatal and postnatal alcohol exposure alters the proportions of different spine shapes in the long (apical) dendrites of hippocampal CA1 pyramidal cells.

Changes in the dendritic morphology and reductions in dendritic spines in the hippocampus interfere with the optimal transmission of the neural impulse. Relating these data to humans, it is possible that these changes could contribute to the cognitive problems seen in children with FAS, because the hippocampus is involved in learning and memory.

### Disrupted Mitochondrial Membrane

The mitochondria are subcellular structures that produce energy for the cell. Changes in the structural integrity of the mitochondrial membrane and its associated proteins have been shown to initiate apoptosis or necrosis, two possible forms of cell death (Kroemer et al. 1997). Ramachandran (2001) demonstrated that alcohol exposure in utero significantly elevates cytochrome *c* and apoptosis-inducing factor (AIF) levels in mitochondria in the fetal rat brain. Light and colleagues (2002) showed that 1 day of alcohol exposure (6.0 g/kg on postnatal day 4) leads to apoptotic Purkinje cells in early generated cerebellar lobules. The observation of these apoptotic neurons coincides with the release of cytochrome *c* from the mitochondria of the Purkinje cells. These studies suggest that alcohol-induced neuronal loss may occur via disruption of mitochondria within those neurons.

### Changes in Cell Adhesion Molecule Properties

The extracellular matrix (ECM), a filamentlike structure on the surface of the cell, is crucial for proper adhesion, migration, and differentiation of neurons in the developing CNS. Therefore, disruptions in the ECM can have severe negative ramifications for developing neurons. One such disruption of the ECM is evident in alcohol’s effects on the L1 cell adhesion molecule (L1CAM), a cell surface protein that promotes cell-to-cell binding. Charness and coworkers (1994) demonstrated that alcohol inhibits the adhesive properties of L1CAM in cell cultures, preventing neurons from properly adhering to each other or to the ECM. Bearer and colleagues (1999) reported that alcohol inhibited L1CAM-mediated outgrowth of the connective fibers from early post-natal rat cerebellar neurons. Collectively, these results suggest that developmental alcohol exposure may alter cellular components, such as L1CAM, and that the consequences of a change in L1CAM function could be detrimental to brain development.

### Neural Circuitry Modifications

A complete, intact, and functional neural circuitry requires proper connections between regions of the brain. Developmental alcohol exposure has been shown to cause aberrant wiring patterns or abnormalities in fiber bundles in the developing brain. For instance, the size of the corpus callosum, one of the largest fiber bundles in the brain, is significantly reduced following prenatal alcohol exposure in rats (Zimmerberg and Scalzi 1989). Further research has shown that, in rats, in utero alcohol exposure alters the distribution of neurons from cortical areas projecting through the corpus callosum into the somatosensory cortex, which receives tactile information from the body (Miller 1997). Recently, Elberger and associates found that alcohol exposure during the period equivalent to the second trimester in rats resulted in an abnormal dendritic branching of corpus callosal projection neurons in the visual cortex (Qiang et al. 2002). These findings demonstrate that the interhemispheric connections are affected by prenatal alcohol exposure. Furthermore, early findings indicated that the topographic organization of hippocampal mossy fibers (neuron extensions that terminate in mosslike branchings) was affected by developmental alcohol exposure (West et al. 1981; West and Hodges-Savola 1983).

Prenatal alcohol exposure also may significantly interrupt the development of the cortical circuitry by interfering with the proliferation and migration of the cortical neurons. Miller (1986, 1993) demonstrated that prenatal alcohol exposure delays the migration of neurons from the zone where they are produced to their final destinations, and the rate of migration of these cortical neurons was decreased as well. Such errors in proliferation and migration disrupt synchronized developmental events, which results in ectopic clusters of cortical neurons and abnormal neuronal circuitry. In sum, because of the critical role of integrated neural circuits within the brain, it is obvious that abnormalities in any component of a neuroanatomical circuit resulting from developmental alcohol exposure would have severe consequences for CNS functions.

## Implications for Prevention and Intervention

Recently, several studies have shown that complex motor training and pharmacological treatments are able to prevent or ameliorate developmental alcohol-induced alterations in specific anatomical structures. These studies are based on the assumption that restoring neuroanatomical integrity would result in subsequent functional recovery. Therefore, identifying the structural alterations associated with prenatal alcohol exposure and understanding the underlying mechanisms of these changes are critical steps in developing both prevention and intervention strategies.

### Complex Motor Training

**Figure 1:**
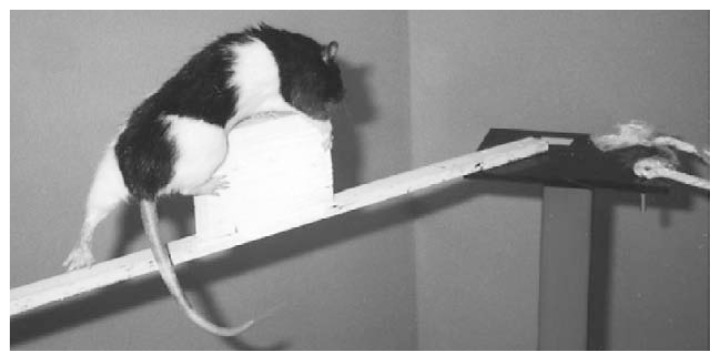
A rat undergoing complex motor training. This training consists of prodding the rat to traverse an elevated course with multiple obstacles and challenges without falling off. Each rat runs the course five times per day for 12 days, and each run is timed. Over time, the rat will learn to traverse the course in less time without falling. Such motor training experiences during adulthood stimulate connections between nerve cells in animals that received alcohol during the early postnatal period (i.e., during the brain growth spurt). The resulting improved performance on the obstacle course suggests a potential intervention that may attenuate the severity of developmental alcohol–induced brain injury. SOURCE: Photograph courtesy of Dr. Anna Klintsova, Binghamton University, Binghamton, New York.

Using a paradigm that involves complex motor training (see figure 1), Klintsova and colleagues (2002) convincingly demonstrated structural reorganization in rat cerebellum following early postnatal alcohol exposure. The most striking and profound aspect of these studies was that, although the complex motor skill training was initiated during adulthood (180 days of age), it was able to successfully stimulate the creation of new connections between Purkinje cells and the long connecting fibers (i.e., parallel fibers) of other neurons in animals that were exposed to alcohol between postnatal days 4 and 9. Although the number of Purkinje cells was unchanged following the complex motor training, the increased volume of the paramedian lobule of the cerebellar cortex and the increased number of parallel fiber synapses per Purkinje cell were sufficient anatomical changes to result in improved motor skills. These findings have relevant clinical significance and important therapeutic implications. They suggest that complex rehabilitative motor training can improve motor performance of children, or even adults, with FAS. However, at present it is unclear whether recovery or reorganization of neural connections is possible in regions other than the cerebellum and motor cortex of the nervous system. Moreover, it remains to be seen whether the improvement in motor skills and anatomical alteration in synapses following complex motor training is permanent.

### Pharmacological Treatments

Antioxidant therapy has been reported to be effective in reducing early postnatal alcohol-induced neurotoxicity in animal studies. Heaton and colleagues (2000) showed that vitamin E protects against early postnatal alcohol-induced cerebellar Purkinje cell loss in lobule I, a lobule that is most sensitive to alcohol during development. Although it has not yet been documented, researchers speculate that antioxidant treatment would protect the functions of the cerebellum from early postnatal alcohol exposure because the antioxidant prevents the formation of harmful free radicals. In contrast to complex motor training, which is an intervention, administering vitamin E during pregnancy is a preventive effort. Prevention in many ways is more effective and economical than intervention in attenuating the detrimental effects of alcohol exposure during development. Recent data show that melatonin, a known and effective antioxidant, when given to neonatal rat pups simultaneously with alcohol, does not prevent the early postnatal alcohol-induced Purkinje cell loss in either the part of the cerebellum known as the cerebellar vermis or in lobule I (Edwards et al. 2002). Taken together, these data suggest that preserving the intact neural structures depends on the type of antioxidant used and possibly the timing of antioxidant administration.

Treatment with antioxidants before alcohol exposure may be critical for the antioxidant to be available in an amount that would most effectively inhibit alcohol-induced generation of free radicals. Recently, two peptides derived from growth factors that are associated with normal development (i.e., NAPVSIPQ [NAP] and SALLRSIPA [SAL, also called ADNF–9]) have been shown to be effective in preventing prenatal alcohol-induced somatic and brain growth retardation in a mouse model (Spong et al. 2001). In addition, supporting evidence indicated that the levels of these peptides were reduced in mouse embryos which were exposed to alcohol prenatally (Spong et al. 2002). More recently, these peptides have been shown to reverse alcohol-induced inhibition of L1-mediated cell-to-cell adhesion in vitro (Wilkemeyer et al. 2002). Despite the promising findings that these peptides prevent some aspects of fetal alcohol-induced damage, their ability to prevent damage to specific neural anatomical structures has not been examined.

Recent data suggest that administering MK–801, a compound that blocks receptors for the brain chemical glutamate, during alcohol withdrawal reduces the loss of hippocampal pyramidal cells in the CA1 region in a neonatal rat model. However, the most profound effect of this pharmacological treatment is its ability to reverse the behavioral impairment in a specific learning task that was induced by early postnatal alcohol exposure (Thomas et al. 2002). These findings demonstrate that preserving the integrity of neural structures is important for upholding function.

**Figure 2:**
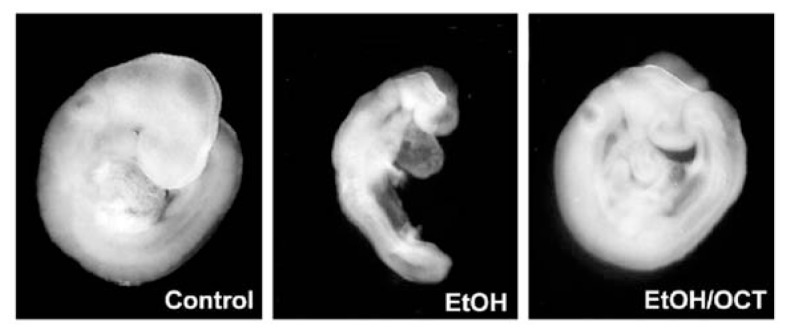
Representative mouse embryos from three experimental treatments. **Left:** Control embryo. **Middle:** Embryo exposed to alcohol (100 mM) in culture for 6 hours. **Right:** Embryo exposed to alcohol (100 mM) and a low dose of the long-chain alcohol l-octanol (3 μM) in culture. It appears that l-octanol treatment significantly reduced the severity of alcohol’s effects on the development of the embryo. SOURCE: Adapted from Chen et al. 2001, with permission.

Further evidence for the effectiveness of certain pharmacological treatments involves the ability of the eight-carbon long-chain alcohol known as 1-octanol to attenuate alcohol-induced damage. Recent data show that a low dose of 1-octanol interacts with the L1CAM to block an alcohol-induced mechanism of abnormal physical development in a mouse embryo model (Wilkemeyer et al. 2000; Chen et al. 2001). As shown in figure 2, it appears that l-octanol significantly reduces the severity of alcohol’s effects. Although it is unclear whether treatment with 1-octanol in vivo would be just as effective as in vitro, or whether 1-octanol protects against alcohol-induced brain damage, this treatment holds promise for developing pharmacological agents to prevent alcohol-induced injury during development. In sum, these anatomical studies of developmental alcohol effects provide opportunities to assess the effectiveness of prevention and intervention methods.

## Conclusion

Identifying specific neuroanatomical anomalies as adverse effects of heavy alcohol exposure during development has been a significant contribution to the fetal alcohol field. Animal studies have been important in the following ways:

- They have demonstrated that alcohol exposure during development, even without polydrug use or undernourishment, can induce significant structural changes to the developing brain.
- They have demonstrated that various regions of the developing brain are not uniformly vulnerable, and that the brain is not equally vulnerable throughout its developmental period. Demonstrating so-called regional and temporal vulnerability, as well as other potential risk factors, has helped researchers to understand the considerable variability observed in children prenatally exposed to alcohol.
- The quantifiable nature of specific neuroanatomical anomalies offers a direct measure for future studies to use in assessing the efficacy of putative neuroprotective agents.

Similarly, important advances also have been made in human studies. Noninvasive MRI studies in children exposed to alcohol in utero have been much more instructive than autopsy studies. They allow neuroanatomical deficits to be correlated with functional deficits. MRI, fMRI, PET, and other neuroimaging techniques offer the potential to help direct future research into pharmacological intervention and treatment strategies with fetal alcohol–exposed patients.

Taken together, human and animal neuroanatomical studies have provided both an experimental foundation for a better understanding of the behavioral impairments associated with heavy maternal drinking, and an important means of evaluating potential neuroprotective and therapeutic interventions.

## Footnotes

1 Heavy drinking is defined as 7 to 13.99 ounces (oz) absolute alcohol per week or 14 to 27.9 standard drinks per week (approximately 12 oz beer, 5 oz wine, 1.25 oz liquor) in the NIH *1988 National Health Interview Survey* (USDHHS 1988).
